# Exploratory analysis of transposable elements expression in the *C. elegans* early embryo

**DOI:** 10.1186/s12859-019-3088-7

**Published:** 2019-11-22

**Authors:** Federico Ansaloni, Margherita Scarpato, Elia Di Schiavi, Stefano Gustincich, Remo Sanges

**Affiliations:** 1 Area of Neuroscience, International School for Advanced Studies (SISSA), Trieste, Italy; 2 Central RNA Laboratory, Istituto Italiano di Tecnologia (IIT), Genova, Italy; 3 Institute of Biosciences and BioResources (IBBR), CNR, Napoli, Italy; 4 Biology and Evolution of Marine Organisms, Stazione Zoologica Anton Dohrn, Napoli, Italy

**Keywords:** Transposable elements, *Caenorhabditis elegans*, Early embryo, Embryogenesis, RNA-Seq, Single-cell, Bioinformatics

## Abstract

**Background:**

Transposable Elements (TE) are mobile sequences that make up large portions of eukaryote genomes. The functions they play within the complex cellular architecture are still not clearly understood, but it is becoming evident that TE have a role in several physiological and pathological processes. In particular, it has been shown that TE transcription is necessary for the correct development of mice embryos and that their expression is able to finely modulate transcription of coding and non-coding genes. Moreover, their activity in the central nervous system (CNS) and other tissues has been correlated with the creation of somatic mosaicisms and with pathologies such as neurodevelopmental and neurodegenerative diseases as well as cancers.

**Results:**

We analyzed TE expression among different cell types of the *Caenorhabditis elegans* (*C. elegans*) early embryo asking *if*, *where* and *when* TE are expressed and whether their expression is correlated with genes playing a role in early embryo development. To answer these questions, we took advantage of a public *C. elegans* embryonic single-cell RNA-seq (sc-RNAseq) dataset and developed a bioinformatics pipeline able to quantify reads mapping specifically against TE, avoiding counting reads mapping on TE fragments embedded in coding/non-coding transcripts. Our results suggest that i) canonical TE expression analysis tools, which do not discard reads mapping on TE fragments embedded in annotated transcripts, may over-estimate TE expression levels, ii) Long Terminal Repeats (LTR) elements are mostly expressed in undifferentiated cells and might play a role in pluripotency maintenance and activation of the innate immune response, iii) non-LTR are expressed in differentiated cells, in particular in neurons and nervous system-associated tissues, and iv) DNA TE are homogenously expressed throughout the *C. elegans* early embryo development.

**Conclusions:**

TE expression appears finely modulated in the *C. elegans* early embryo and different TE classes are expressed in different cell types and stages, suggesting that TE might play diverse functions during early embryo development.

## Background

Transposable Elements (TE) are repetitive elements spread among the genomes of almost all eukaryotes [1]. TE can be classified in transposons and retrotransposons according to their mechanism of transposition. Transposons are composed by DNA and rolling-circle (RC) elements and mobilize through a DNA intermediate, while retrotransposons are composed by Long Terminal Repeats (LTR) and non-LTR (LINE and SINE) sequences that take advantage of an mRNA intermediate for their mobilization [1, 2]. TE make up a large portion of human and murine genomes (40–45%) and despite having been understudied and often considered as *junk* and *selfish* elements, it is currently believed that they have played and continue to play important roles in the biology and evolution of metazoan [2–6]. One of the first observation of the existence and activity of TE was made in *Drosophila melanogaster* where specific outcrosses displayed sterility and other germline abnormalities defined together as hybrid dysgenesis. Further observations lead to the discovery that these phenotypes were due to the lack of silencing, in the specific outcrosses, of the P-element (a DNA transposon) and elucidated the molecular mechanisms causing the phenomenon [7]. Later, Mello and Fire discovered that *Caenorhabditis elegans* (*C. elegans*) mutants, deficient for RNA interference (RNAi), displayed an increased TE mobilization and proposed that the RNAi system has evolved also as a defence response to protect germline from TE activity [8]. Nowadays, although TE activity in the gonads might represent a driving force in genome evolution, it is accepted that it is mostly inhibited by the PIWI/piRNA pathway [9]. Findings in the last decade highlighted that TE mobilization is not confined to germ cells and cancer tissues. They, indeed result expressed and active during embryogenesis [10–17] and even in the adult central nervous system (CNS) [5, 18–23]. TE (mostly LTR) have been proposed to play fundamental roles during embryogenesis, when they shape gene expression acting as regulatory elements, providing promoters and binding sites, regulating chromatin accessibility, and physically interacting with transcripts [10, 11]. Several studies evidenced that TE are needed during mammalian embryogenesis in diverse biological processes such as pluripotency maintenance, embryo viability and immune response priming [12–15]. According to these studies, the complete lack of expression as well as the over-expression of TE is not compatible with the correct development of the mammalian embryo, thus suggesting that the expression of TE is strictly regulated during mammalian embryogenesis. Finally, TE have been suggested to play a dual role in the CNS of organisms such as fruitfly, mouse and human. On one hand, activity of retrotransposons in CNS determines somatic mosaicism [5, 18–23] which has been proposed to be correlated with the evolution of cognitive capabilities [19, 20, 22]. On the other, alteration of their expression and activity have been associated to neurodevelopmental and neurodegenerative disorders [24–27].

*C. elegans* is a ~ 1 mm long nematode largely used as model organism. Its maintenance under laboratory conditions is very simple: the transparent nematode is characterized by a short generation time (3–4 days), its food source is *Escherichia coli* and up to 1000 worms can be cultured at the same time in a 55 mm petri dish [28]. *C. elegans* embryogenesis lasts for ~ 16 h, the embryonic cell lineage has been the first metazoan to be completely mapped in the early eighties and a name has been assigned to all the embryonic cells [29]. In the early stages five asymmetric divisions produce six founder cells: AB, MS, E, C, D, and P4. In more details a P0 zygote gives rise to a larger anterior cell, AB, and a smaller posterior blastomere, P1 (2-cell stage). P1 undergoes an asymmetric division that gives rise to EMS and P2, while AB through a symmetric division gives raise to ABa and ABp (4-cell stage). Subsequent asymmetric divisions of EMS into MS and E, of P2 into C and P3, and symmetric divisions of ABa and ABp, which generate ABal, ABar, ABpl and ABpr, characterize the 8-cell stage. The further divisions of the 8 cells complete the generation of the founder cells whose descendants will produce specific cell types (16-cell stage) [29]. *C. elegans* gene manipulation can be carried out in simple and very effective ways [30, 31]. The adult is composed of about 1000 somatic cells, 302 of which are neurons. Approximately 15% of its genome derives from TE [32]. Unlike fruitfly, mouse and human genomes in which the majority of TE are retrotransposons, *C. elegans* TE are mostly DNA transposons (Additional file 1). Globally, 74% of *C. elegans* TE are annotated as DNA transposons, 16% as RC transposons and 10% as retrotransposons (1% SINE, 4% LINE, 5% LTR). According to literature, the Tc/Mar family (DNA TEs) is the most active, while active retrotransposition was never observed under laboratory conditions [32, 33]. To our knowledge no study has ever been performed on the expression of TE during *C. elegans* development.

Here we explore TE expression dynamics in the *C. elegans* early embryo (from the zygote to the 16-cell stage) taking advantage of the single-cell RNA sequencing (scRNA-seq) dataset generated by Tintori et al. in 2016 [34]. We developed a bioinformatics pipeline aimed at the quantification of TE-specific reads and analyzed *if*, *when* and *where* each specific class of TE is expressed during *C. elegans* development and their potential correlations with the expression of protein coding genes.

## Methods

### Data collection and pre-processing

To study the expression of TE in the *C. elegans* early embryo we took advantage of Tintori et al. scRNA-seq public data [34]. They sequenced single cells from embryos of the 1-, 2-, 4-, 8- and 16-cell stage. Totally, they sequenced 219 cells, generating between 5 and 9 replicates for each of the 31 different cell types. We downloaded raw files containing single-end reads of 50 bp from ENA-EBI database (PRJNA312176 accession code) and discarded 55 samples that did not pass quality filters regarding whole embryo mRNA mass, according to the authors. The filtered dataset is globally composed of 164 samples, each cell type is represented by a minimum of 4 to a maximum of 7 replicates. We report the selected samples in Additional file 2.

### TE expression analysis

**Fig. 1:**
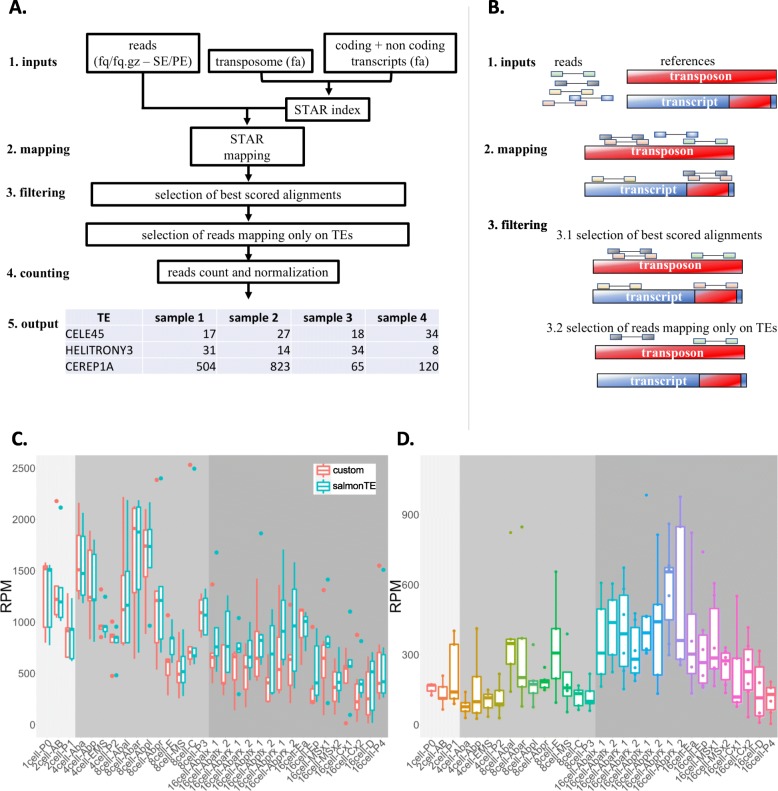
A bioinformatics pipeline for the quantification of read specifically mapping on TE. **a** and **b** Workflow and schema of the pipeline. Reads are mapped, allowing multimapping, against the reference transcriptome (composed by annotated coding and non-coding transcripts [blue] and TE consensus sequences [red]). Best scoring alignments are selected and then, to avoid selection of TE-non-specific reads, reads mapping with best scoring alignments both on transposome and transcriptome are discarded. STAR is the program used for the mapping. **c** Global TE expression levels calculated for every cell type using our pipeline (custom) and SalmonTE. **d** Quantification of TE-non-specific reads used to assess whether the increased TE expression in AB descendant cells of the 16-cell stage, evidenced by SalmonTE, is given by TE-non-specific reads quantification

**Fig. 2:**
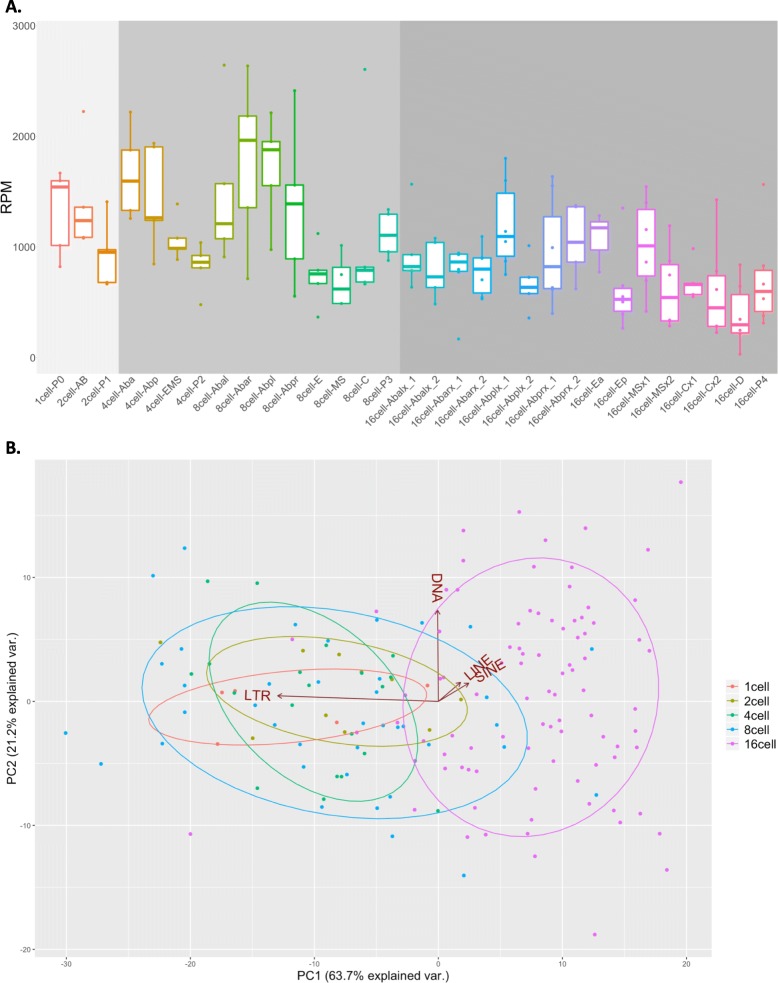
TE global expression profile among the 31 *C. elegans* early embryo cell types. **a** For each analyzed TE, raw read counts have been summed, converted in RPM (y-axis) and plotted for all the 31 *C. elegans* early embryo cell types (x-axis). Cells belonging to 1- and 2-cell stages (light grey background) are transcriptionally inactive and show, together with cells of the 4- and 8-cell stages (grey background), the highest levels of TE expression. TE expression levels decrease in the 16-cell stage (dark grey background) where embryo cell fate starts to be determined. **b** PCA analysis showing the distribution of all the 164 analyzed samples according to their TE expression. The samples can be subdivided in 2 main groups according to their TE expression profiles. The first one is composed by samples belonging mainly to 1-, 2-, 4- and 8-cell stages while the second group is composed by samples belonging to 16-cell stage. LTR expression determines the grouping of 1-, 2-, 4-, 8-cell stages, while non-LTR retrotransposons (SINE and LINE) expression determines the separation of 16-cell stage from the other cell stages. The variance explained by the first two principal components is 63.7 and 21.2% respectively. Three PCs make up 93%, four PCs make up 100% of the TE expression variation

We downloaded the fasta files containing coding (cDNA file – WB235 version) and non-coding transcripts (ncRNA file – WB235 version) from Ensembl (version 93) [35] and the fasta file containing the nucleotidic sequences of all the *C. elegans* repeats from RepBase database [36] which we call the transposome. We discarded from the transposome 16 sequences annotated as Satellite (SAT). We then developed a bioinformatics pipeline (a brief workflow of pipeline rationale is represented in Fig. 1a, b) able to select reads mapping specifically on transposome and not on TE fragments embedded in coding and/or long non-coding transcripts. We used STAR [37] (version 2.6.0c, parameters: --outSAMprimaryFlag AllBestScore, −-outFilterMismatchNoverLmax 0.04) to map the reads on the reference obtained merging the described RepBase transposome and the Ensembl transcriptome files containing coding and non-coding transcripts, assigning primary alignment flag to all the alignments with the best score. Using samtools [38] (v1.3.1) we selected alignments flagged as primary (−F 0 × 100 parameter). To avoid selection of reads mapping on TE fragments embedded in coding and/or long non-coding transcripts we discarded reads mapping with best-scoring alignments both on transposome and transcriptome using custom Python scripts and Picard FilterSamReads tool [39]. Using bedtools coverage [40] (v2.26.0, −counts parameter) we counted for each sample the number of selected reads mapping exclusively on TE. Raw counts have then been normalized on the total number of mapping reads and multiplied by 1000,000 obtaining expression values indicated as reads per million mapped reads (RPM). To test the accuracy of our pipeline we carried out the same analysis using the recent published SalmonTE pipeline [41] that measures TE expression levels using the Salmon tool on RNA-seq data and a set of transposon consensus sequences. First, using the SalmonTE *index* mode (−-te_only parameter) we created the index file starting from the *C. elegans* consensus sequences fasta file downloaded from RebBase database. Then, taking advantage of the SalmonTE *quant* mode (−-exprtype = count parameter) we quantified the TE expression values. Finally, we selected the TE in common between the two analyses and made the comparison between the tools. To perform the principal component analysis (PCA) represented in Fig. 2b we first selected DNA, LTR, LINE and SINE TE and then grouped together the expression values of elements belonging to the same class. The expression values where then transformed using a square root transformation and the PCA analysis was executed using the fast.prcomp() function of the gmodels R library. The first two components were plotted using the ggbiplot() function of the ggbiplot R library.

### TE/gene expression correlation and pathways analysis

We performed a correlation analysis between the expression of the analyzed TE and all the *C. elegans* genes. We retrieved gene expression values (RPKM) from the Supplementary Table S2 of the paper published by Tintori et al. [34]. To select TE and genes with a reproducible expression among the replicates of the same cell type we selected TE and mRNAs with an expression value > = 25 RPM or RPKM in at least 3 replicates of at least 1 cell type. We performed a pairwise correlation analysis between TE and coding genes using Pearson correlation test. Pearson correlation coefficients were calculated using *pearsonr* function of the *scipy* Python module (*stats* sub-module) selecting only correlations with *R*^2^ > = 0.4 or < = − 0.4 and with an FDR (Benjamini & Hochberg) corrected *p*-value <= 0.0001. To analyze potential pathway enrichment for genes involved in the selected correlations, a statistical over-representation test was performed using Panther tool [42] (version: 13.1, reference list: *Caenorhabditis elegans*, Annotation Data Set: Reactome pathways, Test type: Fisher’s Exact with FDR multiple test correction, FDR corrected p-value cut-off < 0.01). All the plots were generated using R Software.

## Results and discussion

### A bioinformatics pipeline to specifically measure TE expression levels

Taking advantage of the scRNA-seq dataset published by Tintori et al [34], we quantified TE expression in all the sampled cells. This dataset is composed of 164 samples subdivided among the 31 different cell types characterizing 5 early embryo cell stages (1-, 2-, 4-, 8- and 16-cell stages). To consider only reads effectively mapping on TE, our pipeline specifically exclude reads mapping on TE fragments embedded in annotated coding and/or long non-coding transcripts. Reads are firstly mapped, allowing multimapping, against a reference transcriptome made of all the annotated transcripts plus the entire species-specific TE consensus sequences from RepBase. Next, for each read, all the alignments with the best score (multimapping reads may have more than one alignment with best score) are selected. Finally, reads aligning with best score exclusively against TE are used for TE expression quantification. This means that a read mapping with the best score against both a TE and a coding/non-coding transcript is discarded (Fig. 1a, b). This strategy avoids the usage of those reads that might derive from TE fragments embedded in annotated transcripts in the measurement of TE expression. In this work, we will call *TE-non-specific* the reads mapping with best alignment score on both a TE and a coding/non-coding transcript and *TE-specific* those reads mapping with the best score exclusively on a TE. On average, about 80% of reads were mapped against the whole reference transcriptome (the union of coding, non-coding and TE transcripts). TE expression resulted low but detectable with a median number of TE-specific reads of 0.1% across all the samples. Interestingly, about 20% of reads mapping with at least one best alignment on TE belongs to the TE-non-specific reads. For these reads it is not possible to determine whether they originated from a coding/non-coding transcript or a TE and therefore, keeping them into account, might cause biased expression level calculations. We carried out the same expression analysis using SalmonTE [41], a recently published tool for TE expression. The results obtained with SalmonTE globally confirmed the general trends observed with our pipeline (Fig. 1c). However, especially in the AB descendant cells of the 16-cell stage, SalmonTE indicated generally higher TE expression levels with respect to our pipeline. To better understand the origin of the difference between the two sets of results, we selected all the TE-non-specific reads and quantified their level for each sample. The results (Fig. 1d) showed that TE-non-specific reads are more abundant in AB-descendant cells (16-cell stage), which correspond to the samples with the highest difference between SalmonTE and our pipeline. These results suggest that the differences observed between the two pipelines are mainly due to the different usage of TE-non-specific reads and that SalmonTE might be using, to measure TE expression levels, also reads which could be deriving from coding/non-coding transcripts. Intriguingly, AB cells of the 16-cell stage give also rise to neurons [34, 43, 44], which are known to be characterized by the expression of a high number of long non-coding RNAs (lncRNAs) which in turn are enriched for TE fragments [45–47]. We therefore believe that the usage of TE-non-specific reads in the quantification of TE expression might lead to an overestimation of TE expression, especially in nervous tissues, caused by the expression of annotated transcripts with embedded TE fragments. Filtering out TE-non-specific reads would lead to a more precise quantification of TE expression.

### TE expression changes among the stages of the *C. elegans* early embryo

The TE global expression profiles in each of the 31 cell type and stage (raw read counts in Additional file 3) is summarized in Fig. 2a and in Additional file 4. It shows that TE abundance is particularly high in the transcriptionally inactive embryo cells (1-cell P0 zygote, 2-cell AB and P1 cells) [48], in the 4-cell stage and in the 8-cell stage. This suggests that TE mRNAs are a component of the maternal mRNAs and are important in the initial developmental stages. A principal component analysis performed on the expression levels of all the *C. elegans* TE belonging to DNA, LTR, LINE and SINE classes (Fig. 2b) shows that the 164 samples could be subdivided in two main groups. The first group mainly collects samples from the initial stages (1-, 2-, 4- and 8-cell stages), while the second group is principally composed by samples from the 16-cell stage. LTR expression determines the grouping of 1-, 2-, 4-, 8-cell stages, while non-LTR retrotransposons (SINE and LINE) expression determines the separation of 16-cell stage from the other cell stages, indicating that these two groups of elements have rather opposite expression dynamics. These results support the observation that LTR and non-LTR retrotransposon expression might be differentially regulated in the *C. elegans* early embryo.

### LTR expression is higher during stages associated to pluripotency maintenance and might activate the embryo innate immune response

**Fig. 3:**
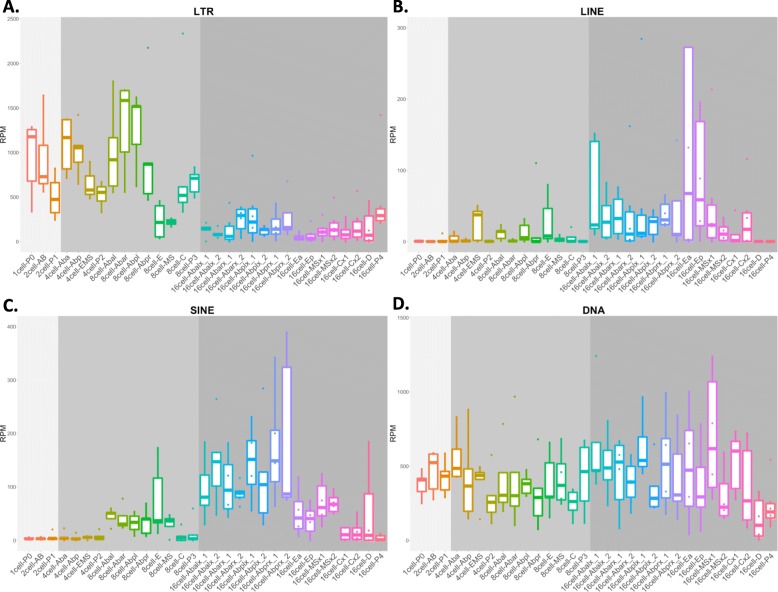
LTR, LINE, SINE and DNA transposon expression in the *C. elegans* early embryo. **a** Expression of LTR. LTR elements are expressed in the initial stages (1-, 2-, 4- and 8-cell stages). **b** Expression of LINE. LINE elements are mostly expressed in E precursor and E descendant cells (EMS cell 4-cell stage, E cell 8-cell stage and Ea and Ep cells 16-cell stage) and in AB descendant cells at 16-cell stage. **c** Expression of SINE. In the *C. elegans* early embryo SINE class is characterized by the expression of a single TE (CELE45) which appears to be specifically expressed in AB descendant cells at the 16-cell stage. **d** Expression of DNA TE. DNA transposons show, as a whole, a constant expression profile throughout the *C. elegans* early embryo analyzed cell types

To better investigate TE classes expression patterns, we separated the TE according to the different classes and inspected their expression levels (Fig. 3). LTR retrotransposons (Fig. 3a) are the elements showing the highest expression levels in the *C. elegans* embryo. Overall, LTR are highly abundant in the initial stages of *C. elegans* embryo development (1-, 2-, 4- and 8-cell stages). In particular, LTR are highly expressed in the zygote (1-cell P0) and in almost all the AB cells of the 2-, 4- and 8-cell stages. Intriguingly, LTR expression decreases strongly in the 16-cell stage. CER1 and LTRCER1 are the two most expressed LTR elements (Additional files 4 and 5). Their expression profiles are very similar and recapitulate general LTR expression profile, since they are both highly expressed in 1-, 2-, 4- and 8-cell stage, while lowly expressed in 16-cell stage. Although the gastrulation process in *C. elegans* begins at the 26-cell stage [49], at the 16-cell stage the fate of all the embryo cells starts to be determined [29, 50] and consequently the number of pluripotent cells drops down. The deep decrease of LTR expression in correspondence of the 16-cell stage may indicate that LTR are mostly expressed in undifferentiated cells, suggesting a role for LTR in the maintenance of pluripotency also in *C. elegans* as already reported in higher organisms. In mouse and human embryonic stem cells (ESCs), different classes of TE are specifically expressed across a transcriptional spectrum of pluripotency [13, 16]. In addition, specific ERVs are re-activated during the reprogramming of somatic cells into induced pluripotent stem cells (iPSCs) [17]. In addition to pluripotency, it has also been shown that LTR-derived nucleic acids may play a role in the activation of innate immune pathways in mammals [51]. *C. elegans* lacks an adaptive immune system, however it does have an innate immune system and is able to respond to external insults from bacteria, fungi and viruses. The *C. elegans* innate immune system is composed by anti-viral and anti-microbial pathways: the anti-viral response is activated by viral double-strand RNA (dsRNA) and is mediated by the RNAi machinery, while the anti-microbial response is composed by different pathways whose induction led to the activation of secreted effector proteins such as C-type lectin anti-microbial peptides (AMPs) [52]. To our knowledge, LTR retrotransposons activity in relation to infections has not been studied in *C. elegans*. However, in higher organisms it has been suggested that LTR elements may have an immuno-protective role triggering the innate immune system and thus activating the embryo to respond to pathogens. Grow et al. studied, in ES cells, the activity of the LTR HERV-K, the most active human endogenous retrovirus family. HERV-K encodes a small accessory protein, Rec, homologous to HIV Rev., which allows nuclear export and translation of viral RNAs. In their work, Rec overexpression resulted in increased expression of viral restriction factors leading the authors to propose that HERV-K might provide an immunoprotective effect for human embryos, activating the innate immune system against different classes of viruses [14].

### LINE elements are mainly expressed in E lineage cells

As shown in the Fig. 3b, LINE are the less expressed class of TE in the *C. elegans* early embryo. Overall, according to our analysis, LINE are expressed in few cell types, mainly belonging to the 16-cell stage. In particular, our results suggest that LINE are expressed in E and E precursor cells (4-cell stage EMS cell, 8-cell stage E cell and 16-cell stage Ea and Ep cells) and, at lower levels, in several AB cells of the 16-cell stage. Intriguingly, the E lineage gives rise to the intestine [34, 53], while AB lineage gives rise to neurons and non-neuronal tissues characterized by high concentration of nervous connections such as pharynx and epidermis [34, 43, 44, 54, 55]. LINE expression in intestine precursor cells was quite unexpected, whereas the expression of LINE in neurons and nervous system associated tissues has already been observed for higher organism [5, 18–23] and will be discussed in the next paragraph. Our analyses evidenced that there is not a single element capable to recapitulate the LINE global expression pattern as resulted for LTR. The general expression profile observed is the sum of different elements showing variable and element-specific expression dynamics. LINE2A and LINE2C1 are mostly expressed in the 4-cell stage EMS cell and in MS cells (16-cell stage), LINE2B is expressed in the 8-cell stage E cell and in the 16-cell stage AB and MSx1 cells while LINE2F, that have an expression of ~ 5-fold with respect to LINE 2A, 2C1 and 2B, seems to be exclusively expressed in Ea and Ep cells of the 16-cell stage (Additional files 4 and 6). This may suggest that different LINE elements might play different roles during *C. elegans* embryogenesis.

### SINE are mainly expressed in AB lineage cells

Figure 3c shows SINE element expression. SINE are expressed at higher levels with respect to LINE, but lower than LTR and DNA transposons. SINE class in the *C. elegans* reference genome is composed of 2 elements (SINE1 and CELE45), with CELE45 being the only one resulting expressed in our analysis (Additional file 4). CELE45 is highly expressed in all the AB cells at the 16-cell stage, suggesting its specific expression in neurons, pharynx and epidermis precursors, as partially shown by LINE. The expression of CELE45 in AB cells at the 16-cell stage seems to be more specific than the expression of LINE and in particular of the LINE2B element. Taken together, our results suggest that both the SINE CELE45 and the LINE LINE2B are expressed in tissues characterized by associations to the nervous system. Expression and activity of non-LTR retrotransposons have already been evidenced in neurons and neuronal precursors in other species. Perrat et al. showed expression and insertional activity of several TE including LINE-like elements in *Drosophila melanogaster* brains [5]. Moreover, several studies proposed that LINE elements are expressed and actively retrotransposed in neuronal precursors during differentiation of the central nervous system inducing somatic mosaicism and increasing the neuronal plasticity in mouse and human brains [18, 23]. Therefore, we speculate that activation of non-LTR elements in *C. elegans* nervous cells during development may play a role in neuronal cell fate specification, leading to neuronal cells diversity and possibly affecting neural plasticity and synapsis formation.

### DNA TE have a heterogeneous expression profiles

DNA TE (Fig. 3d) are expressed at higher levels with respect to SINE and LINE but lower than LTR. DNA transposons are the most abundant TE in the *C. elegans* genome and they are the only class previously suggested to be active in the *C. elegans* genome [32, 33]. Their global expression is relatively constant throughout the analyzed stages and cell types. The most expressed DNA TE are Chapaev1, CEMUDR1, PALTA3, and PALTTTAAA3 (Additional files 4 and 7) and intriguingly these 4 TE have very different profile of expression. Chapaev1 is constantly expressed among the early embryo cell types and its expression recapitulates the overall expression of DNA transposons. CEMUDR1 is expressed in 1-, 2-, 4- and 8-cell stages, its expression profile is similar to the one showed by LTR elements. PALTA3 and PALTTTAAA3 elements are lowly expressed in 1-, 2- and 4-cell stages, their expression increases at 8-cell stage reaching the highest expression in the AB cells of the 16-cell stage. This expression profile is very similar to the one showed by LINE2B and CELE45. These results suggest that DNA transposons have a heterogeneous expression profile that can be divided in the following types: i) constant, ii) LTR like and iii) non-LTR like. DNA transposons are therefore the only TE class constantly expressed in all the cell types of the *C. elegans* early embryo.

### Expression of LTR elements correlates with the expression of genes associated to the innate immune response

**Fig. 4:**
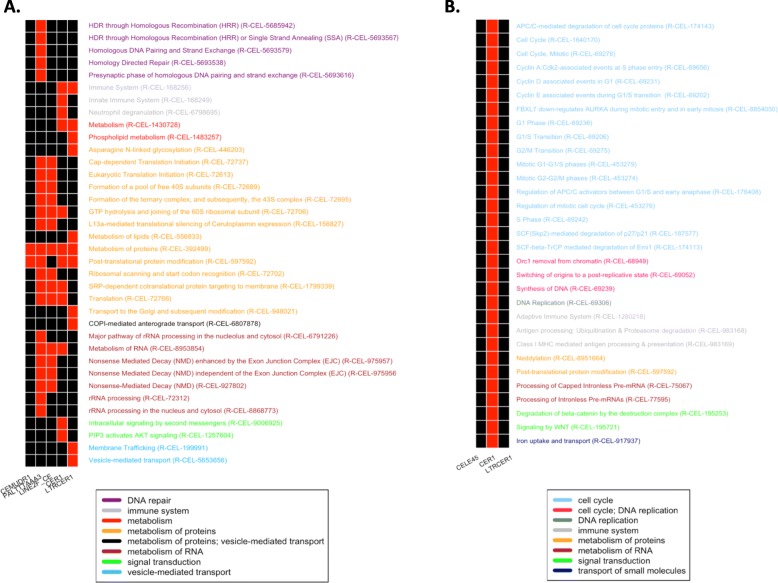
Pathways enriched in genes positively and negatively correlated with TEs. **a** Significantly enriched pathways associated to genes positively correlated with CEMUDR1, PALTTTAAA3, LINE2F, CER1 and LTRCER1. **b** Significantly enriched pathways associated to genes negatively correlated with CER1. (red color means presence, black absence)

In the latest years, several studies reported that, particularly during the embryogenesis, TE may modulate gene expression [11–14]. For this reason, we carried out a correlation analysis between TE and gene expression profiles. Although this analysis does not specifically elucidate any direct interaction between TE and genes, it can highlight similarity in expression profiles that may suggest functional relationships. To perform this analysis, we took advantage of TE expression values calculated using our pipeline and gene expression values calculated in the work published by Tintori et al. [34]. To select TE and genes with reproducible expression levels among replicates of the different cell types we selected TE and genes with expression values higher than 25 in at least 3 replicates of at least 1 cell type. This led to the selection of 11 TE and 6580 genes. We then performed the Pearson correlation test selecting all gene/TE pairs showing an expression correlation with *R*^2^ > = 0.4 or < = − 0.4 and a corrected *p*-value < 0.0001. This resulted in 1300 positively and 169 negatively correlated gene/TE pairs (Additional file 8). The 1300 positive correlations are determined by 1097 non redundant genes: 909 of these are correlated with 1 TE, 173 with 2 and 15 with 3 TE. The 169 negative correlations are determined by a set of 143 non-redundant genes, of which 117 are correlated with 1 TE and 26 with 2 TE. The correlation analysis evidenced that the LTR elements CER1 and LTRCER1, the SINE CELE45 and the DNA CEMUDR determine the highest number of correlations, with CEMUR showing exclusively positive correlations, CELE45, CER1 and LTRCER1 both positive and negative ones (Table 1). We then performed an enrichment analysis to identify pathways associated to genes correlating with TE. This analysis evidenced 66 pathways significantly enriched in the groups of genes determining the identified correlations. Of these, 36 pathways result associated to genes positively correlated with 5 TE (CEMUDR, PALTTTAAA3, LINE2F, LTRCER1 and CER1) (Fig. 4a and Additional file 9) and 31 pathways are associated to genes negatively correlated with a single TE (CER1) (Fig. 4b and Additional file 10). The enriched pathways resulting by positive correlations can be classified in 7 main groups: DNA repair, immune system, metabolism, metabolism of proteins, metabolism of RNA, signal transduction, and vesicle-mediated transport. The enriched pathways resulting by genes negatively correlated with CER1 can be classified in 7 main groups: cell cycle, DNA replication, immune system, metabolism of proteins, metabolism of RNA, signal transduction, and transport of small molecules. Here, it is important to point out that, in some cases, pathway annotations for *C. elegans* might have been inferred by homology, transferring the annotations of homologous genes from more complex species. Results must be therefore interpreted with care considering the specific biological system under investigation. This is especially true for pleiotropic genes, with multiple functions, belonging to multiple pathways in complex organisms. The high number of functions for such genes is likely the result of their evolutionary recruitment into novel biological processes during the route leading to increased organismal complexity. For instance, our analysis identified a significant positive correlation between CER1 and innate immune system genes, a result in agreement with a possible involvement of CER1 in the embryonic activation of the innate immune response in *C. elegans*. On the other hand, this element also results negatively correlated with genes associated to the adaptive immune system, which is unlikely as *C. elegans* does not possess an adaptive immune response. However, these same genes are also annotated as belonging to the ubiquitination pathway, a function consistent with the biological system under analysis. Taking all this into account, we believe that our correlation analysis supports the conclusion that genes associated to the innate immune response are significantly enriched among those whose expression correlates with the expression of LTR elements, reinforcing our previous observations.

**Table 1:** Number of positive and negative correlations for the 11 selected TEs

TE	Class	Correlations	Positive corr.	Negative corr.
CEMUDR1	DNA	202	202	0
CEREP1A	DNA	48	48	0
Chapaev-1	DNA	4	4	0
PALTA3	DNA	96	96	0
PALTTTAAA3	DNA	104	104	0
TC5	DNA	8	8	0
LINE2F	LINE	77	77	0
CER1	LTR	363	323	40
CER3–1	LTR	84	84	0
LTRCER1	LTR	286	248	38
CELE45	SINE	197	106	91

1st column: list of the 11 TE with RPM > 25 in at least 3 replicates of at least 1 cell type. 2nd column: TE classes (DNA, LINE, SINE, LTR). 3rd column: total number of correlations between TE and genes with RPKM > 25 in at least 3 replicates of at least 1 cell type. 4th column: number of positive correlations. 5th column: number of negative correlations

## Conclusions

Several studies have recently reported the expression of TE in mammalian embryos and the CNS suggesting their role in fundamental biological processes such as pluripotency maintenance, embryo viability and differentiation, brain functioning, evolution and diversification [2, 12–14, 18–22]. In this study we developed a bioinformatics pipeline able to quantify reads specifically mapping on TE and explored TE expression in the *C. elegans* early embryo, from zygote to 16-cell stage. Our results suggest that, especially in neural tissues, a portion of reads mapping on TE cannot be distinguished by reads deriving from TE fragments embedded in annotated transcripts. These non-specific reads should therefore be discarded to avoid biases in the estimation of TE expression. In addition, our data show that TE are expressed in the *C. elegans* embryo and that, despite their low level of expression, they present different expression profiles in different embryonic stages and cell types, suggesting a specific regulation during early development. We observed a clear split of developmental TE expression levels in two phases characterized by the expression of two different families of TE, LTR and non-LTR. LTR elements resulted to be mostly expressed in the initial stages (1-, 2-, 4-, 8-cell stages). In particular, according to timing and territories of expression we propose that LTR expression (mainly LTRCER1 and CER1 elements) in the initial developmental stages might play a role in the maintenance of pluripotency and/or the innate immune response activation. We also observed that LINE are mostly expressed in intestine precursor cells (E lineage) and, together with CELE45 (SINE), in 16-cell stage AB cells, the ones giving rise to neurons and tissues connected with nervous system. These results are consistent with the observations reporting the expression of non-LTR elements in nervous tissues of other organisms like fruitfly, mouse and human [5, 18–23]. DNA transposons are the most abundant TE fixed in the *C. elegans* genome and, according to our results, the only TE class expressed in all the cell types of the *C. elegans* early embryo. Overall, DNA transposons are constantly expressed and are composed by TE with heterogeneous expression profiles that can be summarized in: i) constant (Chapaev1), ii) LTR-like (CEMUDR1) and iii) non-LTR-like (PALTA3 and PALTTTAAA3).

To our knowledge this is the first report analyzing expression of TE in the *C. elegans* early embryo and no work on the effects of TE silencing during the *C. elegans* development has ever been performed. In this work we have tried to support our speculations reasoning at a broader evolutionary context, taking into account experiments made in other organisms. Experiments of TE silencing in developmental and/or cellular contexts have been performed mainly in cultures of mammalian pluripotent stem cells and no comprehensive inspections on the effects of TE silencing during whole development of an entire embryo have been reported. In 2004 Park and colleagues [56] silenced, in the mouse zygote, the MT transposon like element, which belong to the LTR family and is expressed in the oocyte. The silencing resulted in the block of the zygote division thus suggesting a fundamental role played by a transposon during mouse embryogenesis. Lu et al. [57] silenced the LTR retrotransposon HERVH in hESC and observed a morphological change with cells adopting a fibroblast-like appearance. Furthermore, they also described a significant up-regulation of HERVH during the reprogramming of fibroblasts into induced pluripotent stem cells (iPSCs) supporting the involvement of the HERVH retrotransposon in the maintenance of the pluripotency state in hESCs. Future RNAi experiments of TE in *C. elegans* embryos might validate whether the expression of TE has any functional role. Departing from our results, a first indicative experiment would consist in silencing the most expressed LTR, LTRCER1 and CER1, followed by measuring the embryo susceptibility to viral and bacterial attacks and its capability to correctly develop and differentiate.

We propose that, despite the low level of expression, TE transcription is finely regulated during the early embryo development of *C. elegans* and might be involved in specific developmental functions in agreement and reinforcing what has already been observed in more complex organisms.

## Supplementary information

**Additional file 1. **TE occupancy in *C. elegans*, *Drosophila, mouse and human* reference genomes. Genomic occupancy of the 5 TE classes (DNA – light red, RC - ochre, LINE – light green, LTR - cyan and SINE - purple) in *C. elegans*, *D. melanogaster*, *M. musculus* and *H. sapiens* genomes. More than 70% of *C. elegans* TE are DNA transposons, ~ 60% of *Drosophila* TE are annotated as LTR and intriguingly *Drosophila* genome completely lacks SINE, ~ 40% of mouse TE are annotated as SINE and ~ 45% of human TE are LINE. TE annotation files for the 5 species have been retrieved from UCSC database (https://genome.ucsc.edu).

**Additional file 2. **SRA accession ID for the 164 analyzed samples. For each of the 31 *C. elegans* early embryo cell type is reported the NCBI-SRA database accession ID of the replicates that passed the quality filters regarding whole embryo mRNA mass according to the authors [34]. 1st column contains the names of the 31 cell types, the 2nd contains the SRA ID of each replicate separated by commas.

**Additional file 3. **Raw read counts of the 163 *C. elegans* analyzed TE. Table containing the raw read counts measured with our pipeline. 1st column contains the names of the 163 analyzed TE. 2nd column contains the classes (DNA, LTR, LINE, RC, SINE) of the TE. Columns 3rd-166th contain the raw read counts for every sample measured with our pipeline.

**Additional file 4. **TE expression profiles in the *C. elegans* early embryo. For each TE belonging to DNA, LTR, LINE and SINE classes (rows) are reported the log_10_(RPM) expression values of each replicate of the 31 cells analyzed (columns). Black color means no expression, green low and red high expression. The horizontal color band above the picture corresponds to different stages (1-, 2-, 4-, 8- and 16-cell stages – left to right), while the vertical color band on the left side of the picture indicates the TE classes (DNA, LTR, LINE, SINE – top to bottom).

**Additional file 5. **CER1 and LTRCER1 expression profiles in the *C. elegans* early embryo. A) CER1 and B) LTRCER1 expression profiles. CER1 and LTRCER1 are the most expressed LTR elements. The two LTR are expressed in the 1-, 2-, 4- and 8-cell stages while their expression in the 16-cell stage is very low. Their expression profiles recapitulate the global expression pattern of LTR elements.

**Additional file 6.** Expression profiles of the 4 most expressed LINE. A) and C) LINE2A and LINE2C1 have similar expression profiles and are mostly expressed in EMS cell (4-cell stage) and in MSx2 cell (16-cell stage). B) LINE2B is expressed in 8-cell E, in AB cells and in MSx1 cell of the 16-cell stage. D) LINE2F is expressed ~ 5-fold with respect to LINE 2A, 2B and 2C1 and its expression seems to be related to 16-cell stage Ea and Ep cells.

**Additional file 7.** Expression profiles of the 4 most expressed DNA transposons. A) CEMUDR1 has an LTR-like expression profile: it is expressed in the 1-, 2-, 4- and 8-cell stages and not in the 16-cell stage. B) Chapaev-1 has a constant expression profile that recapitulates the general DNA transposon class profile of expression. C) and D) PALTA3 and PALTTTAAA3 have a non-LTR-like profile of expression: these TE are mostly expressed in 16-cell stage AB cells.

**Additional file 8. **Significant correlations between TE and genes. Table containing the 1469 correlations with *R*^2^ > = 0.4 or < = − 0.4 and FDR corrected *p*-value < 0.0001 between the 11 selected TE (expression value > 25 RPM in in at least 3 replicates of at least 1 cell type) and the 6580 selected genes (expression value > 25 RPKM in in at least 3 replicates of at least 1 cell type). 1st column contains TE names, 2nd gene names, 3rd R^2^ score, 4th *p*-value and 5th FDR corrected *p*-value.

**Additional file 9. **Enriched pathways associated to genes positively correlated with TE. 1st column contains significantly enriched Reactome pathways associated to genes positively correlated with TE, evidenced by Panther tool with an FDR corrected *p*-value < 0.01. Columns 2nd-6th contain absence (0) or presence (1) of the pathways in CEMUDR1, PALTTTAAA3, LINE2F, CER1, LTRCER1. 7th column contains FDR corrected *p*-value and 8th the pathway classification.

**Additional file 10. **Enriched pathways associated to genes negatively correlated with TEs. 1st column contains over-represented Reactome pathways associated to genes negatively correlated with TE, evidenced by Panther tool with an FDR corrected *p*-value < 0.01. Columns 2nd-4th contain absence (0) or presence (1) of the pathways in CELE45, CER1 and LTRCER1. 5th column contains FDR corrected *p*-value and 6th the pathway classification.

## Data Availability

The datasets analyzed during the current study are available in the ENA EBI repository (PRJNA312176) https://www.ebi.ac.uk/ena/data/view/PRJNA312176.

## Abbreviations

*C. elegans*: *Caenorhabditis elegans*
CNS: Central nervous system
ESCs: Embryonic stem cells
iPSCs: Induced pluripotent stem cells
LINE: Long interspersed nuclear elements
lncRNAs: Long non-coding RNAs
LTR: Long terminal repeats
RC: Rolling-circle
RNAi: LTR RNA interference
RPM: Reads per million mapped reads
sc-RNAseq: Single-cell RNA-seq
SINE: Short interspersed nuclear elements
TE: Transposable elements
