# Supplementary figures and images for: Exploratory analysis of transposable elements expression in the C. elegans early embryo
Source: BMC Bioinformatics. 2019 Nov 22;20(Suppl 9):484. doi: 10.1186/s12859-019-3088-7 (PMC6873666; doi:10.1186/s12859-019-3088-7)

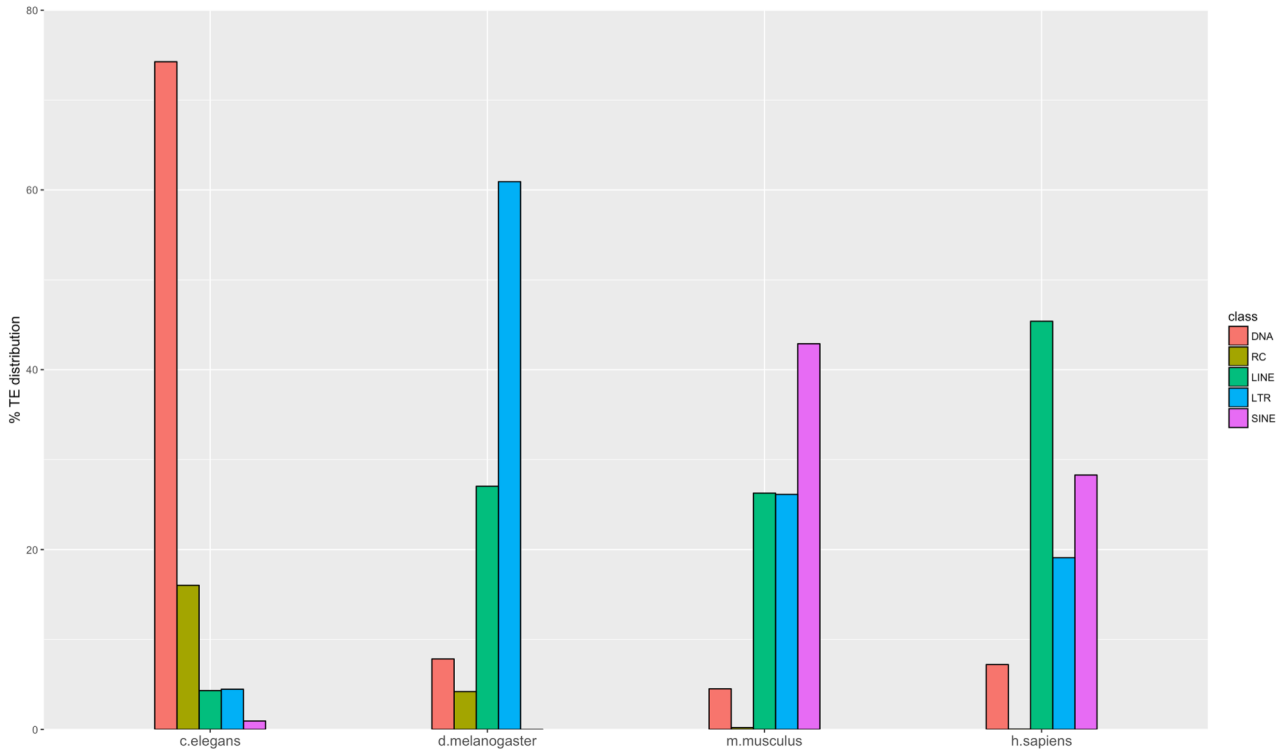

Supplement: Supplementary file 1 — Additional file 1. TE occupancy in C. elegans, Drosophila, mouse and human reference genomes. Genomic occupancy of the 5 TE classes (DNA – light red, RC - ochre, LINE – light green, LTR - cyan and SINE - purple) in C. elegans, D. melanogaster, M. musculus and H. sapiens genomes. More than 70% of C. elegans TE are DNA transposons, ~ 60% of Drosophila TE are annotated as LTR and intriguingly Drosophila genome completely lacks SINE, ~ 40% of mouse TE are annotated as SINE and ~ 45% of human TE are LINE. TE annotation files for the 5 species have been retrieved from UCSC database (https://genome.ucsc.edu). [file 12859_2019_3088_MOESM1_ESM.pdf]

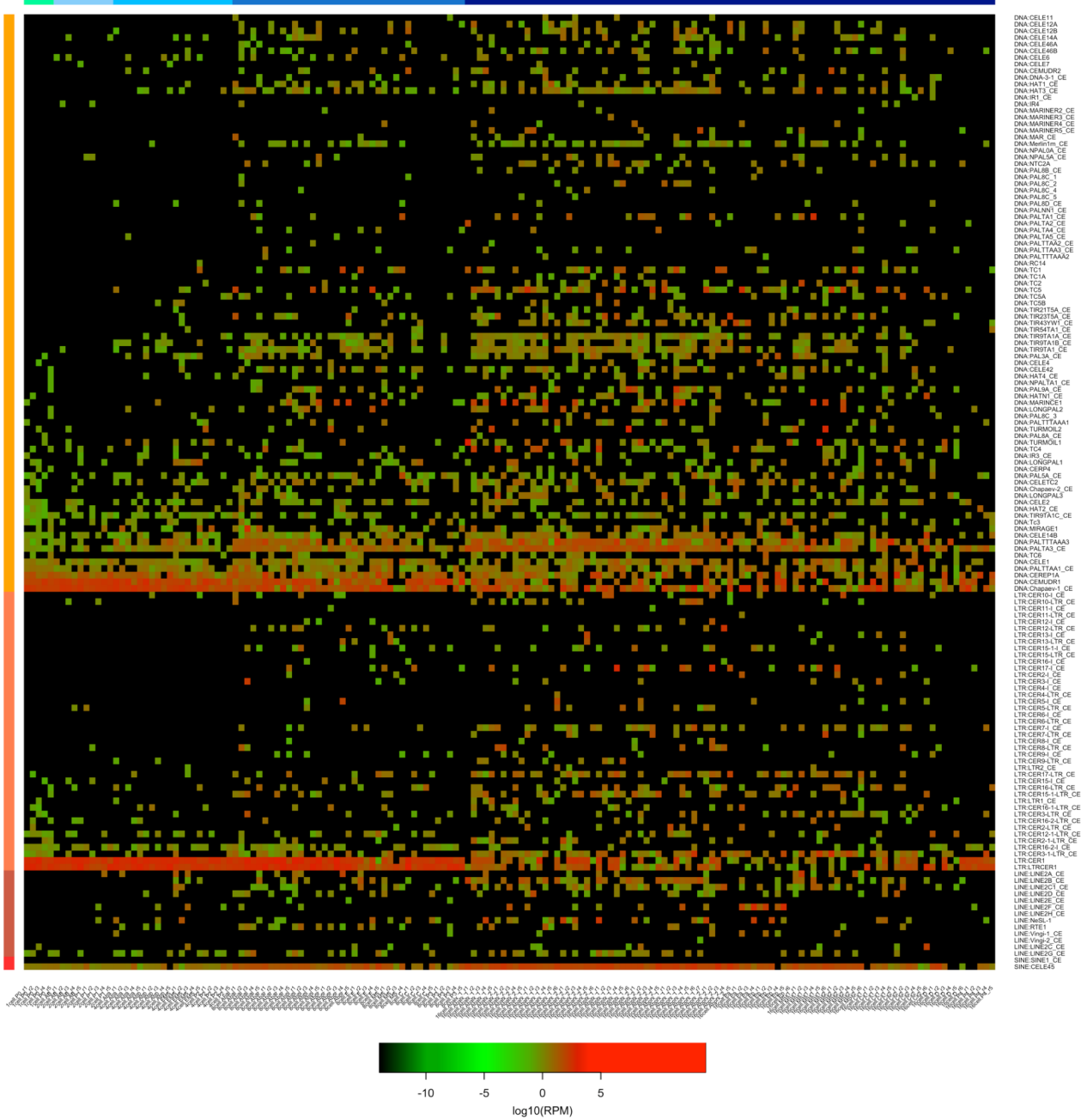

Supplement: Supplementary file 4 — Additional file 4. TE expression profiles in the C. elegans early embryo. For each TE belonging to DNA, LTR, LINE and SINE classes (rows) are reported the log10(RPM) expression values of each replicate of the 31 cells analyzed (columns). Black color means no expression, green low and red high expression. The horizontal color band above the picture corresponds to different stages (1-, 2-, 4-, 8- and 16-cell stages – left to right), while the vertical color band on the left side of the picture indicates the TE classes (DNA, LTR, LINE, SINE – top to bottom). [file 12859_2019_3088_MOESM4_ESM.pdf]

**A.****CER1-LTR**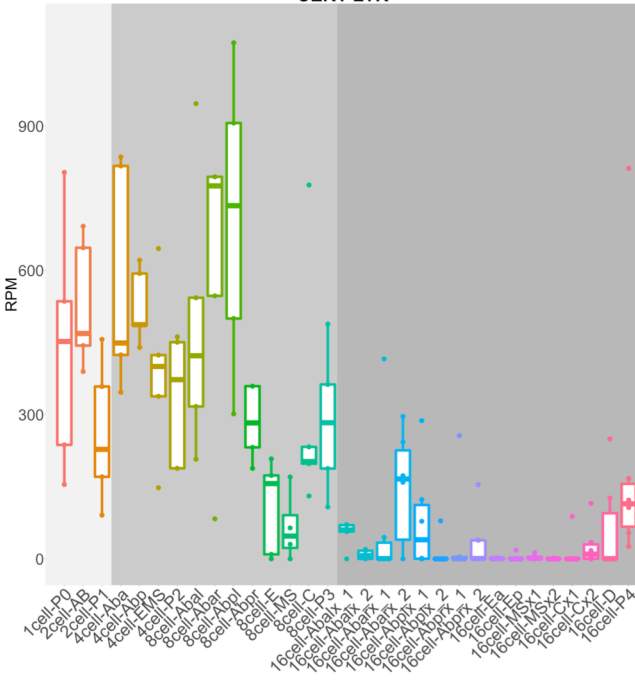**B.****LTRCER1-LTR**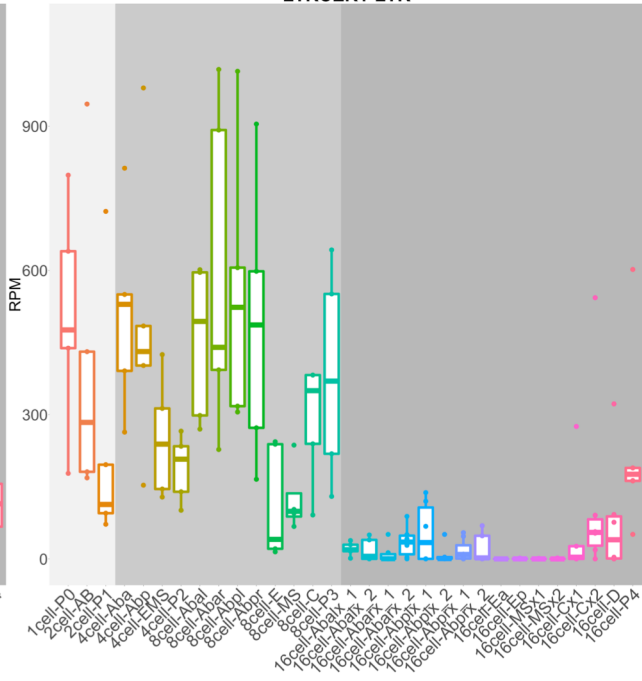

Supplement: Supplementary file 5 — Additional file 5. CER1 and LTRCER1 expression profiles in the C. elegans early embryo. A) CER1 and B) LTRCER1 expression profiles. CER1 and LTRCER1 are the most expressed LTR elements. The two LTR are expressed in the 1-, 2-, 4- and 8-cell stages while their expression in the 16-cell stage is very low. Their expression profiles recapitulate the global expression pattern of LTR elements. [file 12859_2019_3088_MOESM5_ESM.pdf]

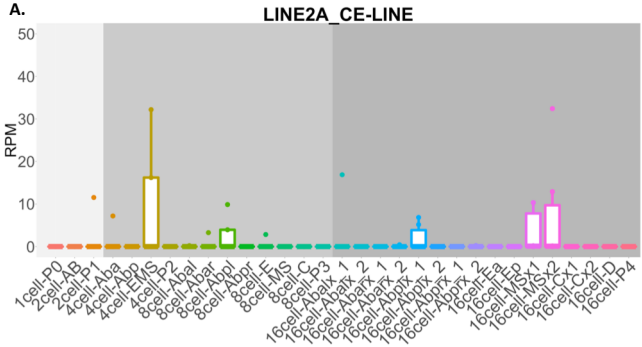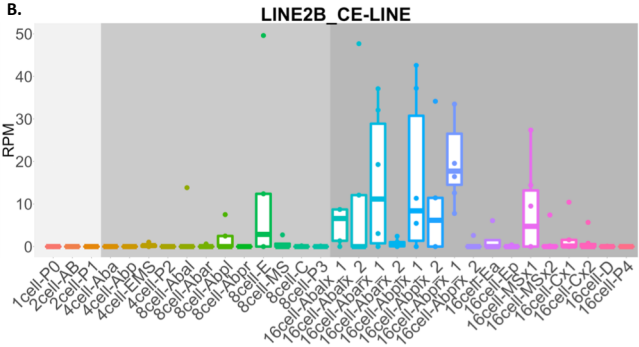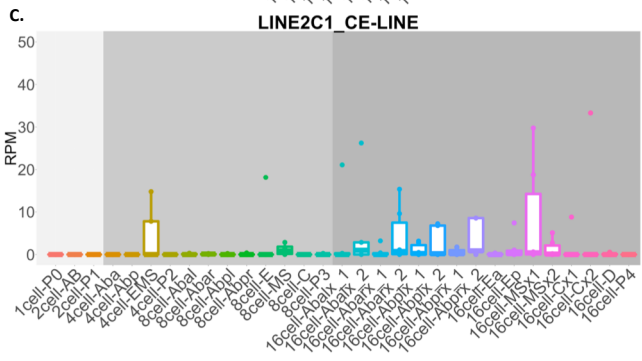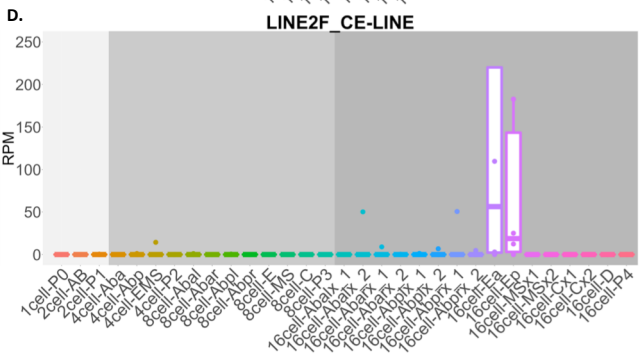

Supplement: Supplementary file 6 — Additional file 6. Expression profiles of the 4 most expressed LINE. A) and C) LINE2A and LINE2C1 have similar expression profiles and are mostly expressed in EMS cell (4-cell stage) and in MSx2 cell (16-cell stage). B) LINE2B is expressed in 8-cell E, in AB cells and in MSx1 cell of the 16-cell stage. D) LINE2F is expressed ~ 5-fold with respect to LINE 2A, 2B and 2C1 and its expression seems to be related to 16-cell stage Ea and Ep cells. [file 12859_2019_3088_MOESM6_ESM.pdf]
