# Supplementary material for: Exploratory analysis of transposable elements expression in the C. elegans early embryo
Source: BMC Bioinformatics. 2019 Nov 22;20(Suppl 9):484. doi: 10.1186/s12859-019-3088-7 (PMC6873666; doi:10.1186/s12859-019-3088-7)
Supplement: Supplementary file 7 — Additional file 7. Expression profiles of the 4 most expressed DNA transposons. A) CEMUDR1 has an LTR-like expression profile: it is expressed in the 1-, 2-, 4- and 8-cell stages and not in the 16-cell stage. B) Chapaev-1 has a constant expression profile that recapitulates the general DNA transposon class profile of expression. C) and D) PALTA3 and PALTTTAAA3 have a non-LTR-like profile of expression: these TE are mostly expressed in 16-cell stage AB cells. [file 12859_2019_3088_MOESM7_ESM.pdf]

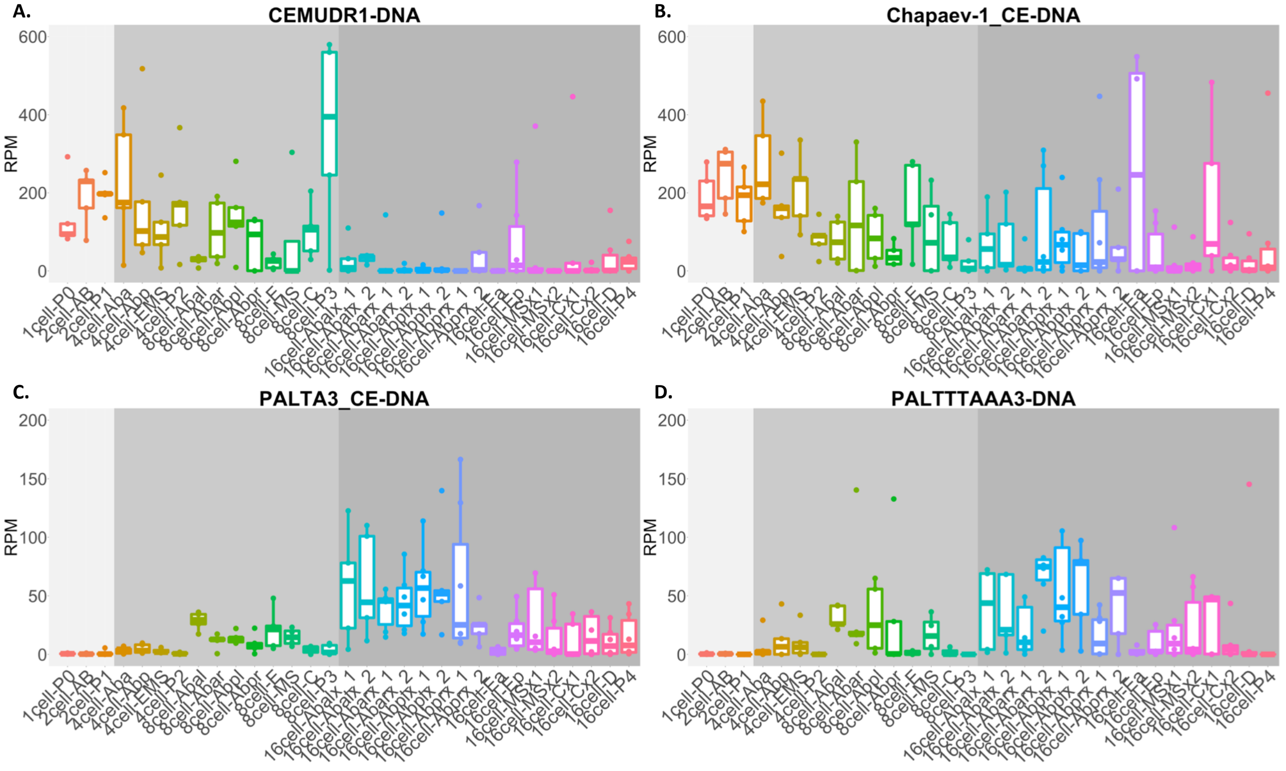
A.
CEMUDR1-DNA
RPM
0
200
400
600
B.
Chapaev-1_CE-DNA
C.
PALTA3_CE-DNA
RPM
0
50
100
150
200
D.
PALTTTAAA3-DNA
1cell-P0
2cell-AB
2cell-P1
4cell-Aba
4cell-Abp
4cell-EMS
4cell-P2
8cell-Abal
8cell-Abar
8cell-Abpl
8cell-Abpr
8cell-E
8cell-MS
8cell-C
8cell-P3
16cell-Abalx 1
16cell-Abalx 2
16cell-Abarx 1
16cell-Abarx 2
16cell-Abplx 1
16cell-Abplx 2
16cell-Abprx 1
16cell-Abprx 2
16cell-Ea
16cell-Ep
16cell-MSx1
16cell-MSx2
16cell-Cx1
16cell-Cx2
16cell-D
16cell-P4
